# Decentralized Opportunistic Spectrum Resources Access Model and Algorithm toward Cooperative Ad-Hoc Networks

**DOI:** 10.1371/journal.pone.0145526

**Published:** 2016-01-04

**Authors:** Ming Liu, Yang Xu, Abdul-Wahid Mohammed

**Affiliations:** School of Computer Science and Engineering, University of Electronic Science and Technology of China, Chengdu, Sichuan, P.R. China; Politehnica University of Bucharest, ROMANIA

## Abstract

Limited communication resources have gradually become a critical factor toward efficiency of decentralized large scale multi-agent coordination when both system scales up and tasks become more complex. In current researches, due to the agent’s limited communication and observational capability, an agent in a decentralized setting can only choose a part of channels to access, but cannot perceive or share global information. Each agent’s cooperative decision is based on the partial observation of the system state, and as such, uncertainty in the communication network is unavoidable. In this situation, it is a major challenge working out cooperative decision-making under uncertainty with only a partial observation of the environment. In this paper, we propose a decentralized approach that allows agents cooperatively search and independently choose channels. The key to our design is to build an up-to-date observation for each agent’s view so that a local decision model is achievable in a large scale team coordination. We simplify the Dec-POMDP model problem, and each agent can jointly work out its communication policy in order to improve its local decision utilities for the choice of communication resources. Finally, we discuss an implicate resource competition game, and show that, there exists an approximate resources access tradeoff balance between agents. Based on this discovery, the tradeoff between real-time decision-making and the efficiency of cooperation using these channels can be well improved.

## Introduction

Communication resources always play a latent role in networked large-scale agent team coordination applications, such as multi-robots system, mobile sensor system, etc. With the expansion of the system, communication resources exert a momentous impact on the cooperative efficiency [1], and numerous attention from both industry and academia has been devoted to this research [2]. For instance, the utmost transfer rate of IEEE 802.11b protocol is 11Mbit/s, and with the insecurity of latency and packet loss, this may fail to meet the capacity requirement of large-scale robots carrying video equipment for surveillance in an open environment [3]. In our previous work [4], we found that, with the expansion of the team size, robots will compete the limited spectrum resources, which is a phenomenon also supported by other studies [5, 6]. On the other hand, different from Cognitive Radio (CR) [7], Mobile Sensor [8] and other traditional wireless communication researches, multi-agent system usually consist of multiple inexpensive agents, and without a strong central processing unit or resources pre-authorization, but with more incomplete channels observation and changing dynamics. In addition, there are no typical technical characteristics, such as a fixed base station or a central node to manage and distribute channels, etc. The major communication mode for most decentralized multi-agent system is Ad-hoc network [2]. However, the typical pre-authorization and consultative allocation approach cannot be applied in the dynamic tasks and agents’ migration. In consequence, new concepts and strategies should be developed, and this is the main motivation proposed here.

As a main technical part of our research. In this paper, we model the decentralized multi-agent multi-channel access problem as a Decentralized Partially Observable Markov Decision Process (Dec-POMDP) problem. We use a continuous time Markov model to simulate the usage of channels while the constant slotted opportunity is used to support agents’ interaction. In addition, we use a sample-based Partially Observable Markov Decision Process (POMDP) to simplify the model. Finally, based on game theory, we model and analyze implicit resource competition between agents, and prove the existence of equilibrium in an ideal state.

## State of the Art

Even though centralized channel resource allocation methods can provide some sort of optimal solutions, they are less effective in situations where the central point fails. For instance, typical auction-based algorithms generally have low communication requirements [9], and the negotiation process, in addition, can degrade in overall efficiency as communication deteriorates [10]. It has been shown in [11] that spatial channels opportunity allocation is equivalent to a graph coloring problem, which objective is to obtain colors assignment that maximizes the utility. But obtaining the optimal coloring is generally known to be NP-hard.

Opportunistic Spectrum Access (OSA) [12] and Opportunistic Spectrum Sharing (OSS) [13] are widely adopted in most recent researches, and several investigations have modeled OSA problems as a POMDP model [14]. Basic OSA concept is described as an agent, which can identify and access idle frequency bands and obtain maximized rewards. Many decentralized methods have referenced the design of POMDP, varying reliance on schemes and can only handle intermittent communication resource scheduling. Reinforcement learning (RL) [15] is a paradigm to solve POMDP problems, and it is inspired by a learning theory which has good performance in multi-robots decision applications [16, 17]. For most RL-based multi-agent systems, the rewards are more achieved by long-team learning, which is the expected accumulated reward that the agent expects to receive in the future under the policy, and can be specified by update value function. However, for the fixed utility function design, time restrains, interaction and observation limited applications, RL is restricted.

Game Theory provides another approach to OSA. Stochastic game [18] as an extension of Game Theory, can improve the capability to solve the OSA problems, and a deeper analysis between the game and the graph-based method is noted in [19]. It is important to note that in many situations, states of the system cannot be observed completely. Therefore, some researches adopt the definition of Partially Observable Stochastic Game (POSG), and a cooperative case of POSG, namely Dec-POMDP [20]. Although some efforts have been made in building heuristic algorithms to solve this intrinsic NEXP-complete problem [21], it is still less feasible obtaining optimal results in a limited time with the partial observation over channels. In addition, in a non-cooperative case, this Dec-POMDP will no longer be suitable.

Many existing works assume that the observation information obtained from an agent’s neighbors is highly correlated. It can improve the efficiency of multi-agent coordination. In this case, exchange of local observations becomes important in coordination. From this view, we present a decentralized cooperative game model in which agents can iteratively adapt their strategies in terms of reduced competition or conflict, and can meet the minimum communication requirements for each agent timely. This presents a novel approach addressing the gaps in the aforementioned works.

## System Model and Problem Statement

In this section, we follow the basic idea of continuous time Markov model to define the basic model of a multi-Channel access problem, and then describe the specific functional definition of each variable and the decision model.

### Multi-Channel Access Model

We consider a multi-agent Ad-hoc network as being created by agents themselves in an open environment, with set $R=\{r_{1},r_{2},....r_{N}\}$ consisting of *N* distributed agents. Although the multi-hops information sharing method can make each agent finally gain full knowledge of the global state, this consumes a lot of communication resources and also deteriorates the system’s performance. Therefore, an agent makes decisions based on its limited observations, and the entire system would still be partially observed. The network consists of a set of contiguous, orthogonal (non-interfering) and homogeneous channels (e.g., 3 such channels in IEEE 802.11b/g and 12 in IEEE 802.11a), denoted by *CH* = {*c*_1_, *c*_2_, …, *c*_*K*_}. The available channels are also numbered from 1 to *K*, and we assume that *N* > *K* agents are seeking channel opportunities in these *K* channels.

**Fig 1:**
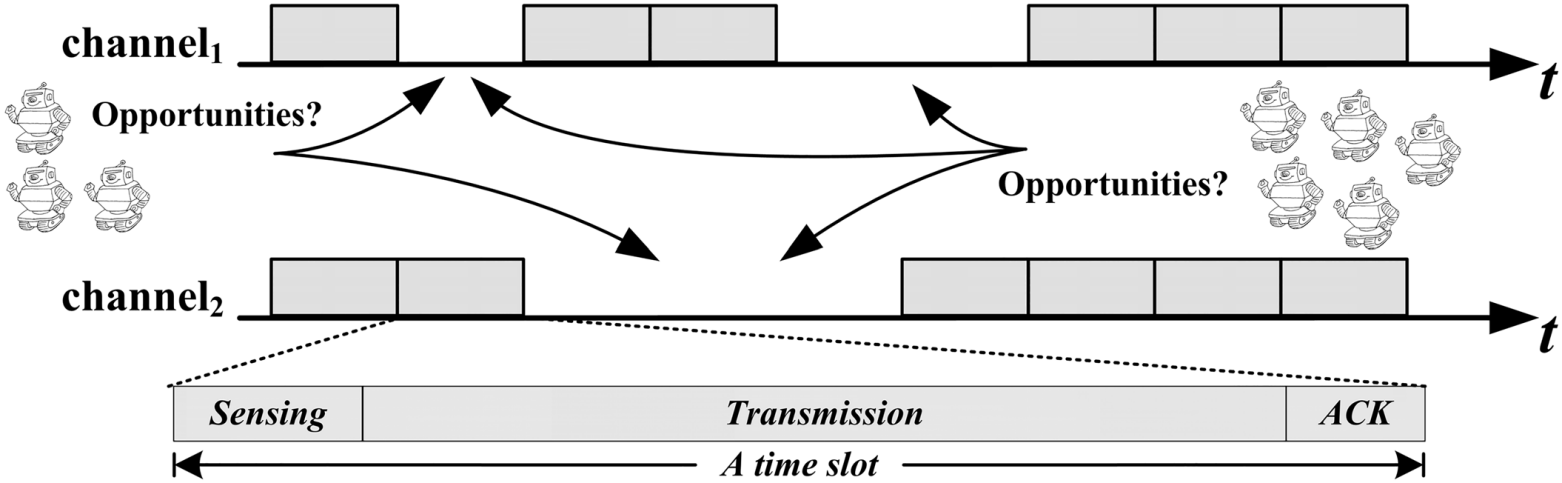
Multi-agent Multi-channel Access Opportunities. Several agents are independently seeking available communication opportunities in two channels, a suitable mechanism is required to ensure smooth communication and low conflicts.

We should recognize that agents can only access channels if the sensed channels are idle. As shown in Fig 1, a time slot consists of 3 parts: sensing, transmission and acknowledgment. Because of practical considerations, agent *r*_*i*_ can sense a set of channels and a subset of sensed channels to access. Limited by its hardware constraints, *r*_*i*_ can sense {*C*_1_} channels, ({*C*_1_} ∈ {*CH*}, |*C*_1_ | <*K*) channels and access {*C*_2_} channels, ({*C*_2_} ∈ {*C*_1_}, |*C*_2_| < |*C*_1_|) channels. State statistics of the *K* channels follows a discrete-time Markov process with 2^*K*^ states, where state is either *idle* or *occupied*. The channel sensing and access decisions are made to maximize agents reward by fully exploiting the sensing of vacant opportunities and the history statistics.

### Multi-channel Access Decision Problem

For some reasons, some agents change the status of stable channel access(switch to other channels, increasing the data flow or strong interference, etc.), and other agents need to adjust the channel access based on their limited observation so as to ensure the global rational use of resources and QoS. Therefore, the multi-agent multi-channel access problem can be described as cooperative searching for available resources in a partially observable multi-channel network. As such, this can be modeled as a Dec-POMDP problem in terms of interdependence. A finite-horizon Dec-POMDP can be defined as a tuple $<S,\Lambda ,T,\Omega ,O,R,p_{0}>$, where

- *S* = {*s*_0_, *s*_1_, …*s*_*n*_} denotes the finite set of network states.
- *A*_*i*_ = {*a*_1_, *a*_2_, …, *a*_*m*_} denotes *r*_*i*_’s available actions set. At each time step, all the agents in R take a joint action $\Lambda^{t}=\times_{1\leq i\leq m}\left\{a_{i}^{t}\right\}$.
- *T* denotes Markovian state transition function. *P*(*s*′|*s*, Λ^*t*^) denotes the probability that doing action Λ^*t*^ and being in state *s* then going to state *s*′.
- $\Omega^{t}=\times_{1\leq i\leq k}\left\{\omega_{i}^{t}\right\}$ denotes the set of joint observations of all the agents and $\omega_{i}^{t}$ is the observation by *r*_*i*_ at time *t*.
- *O* denotes observation function, which specifies the probability of joint observation *O*(Ω^*t*^|*s*′, Λ^*t*^).
- $R(s^{\prime }|\Lambda^{t},s)$ denotes the reward value obtained from taking action Λ^*t*^ in state *s*.
- *p*_0_ = {*B*^0^, *s*_0_} is the initial belief and state distribution.

Action *a* is determined by the policy *π*: *b*→*a*, which is the function that maps a belief state to the action that an agent should execute. $\Omega_{i}^{t-1}$ = ×_1 ≤ *i* ≤ *t*−1_{*ω*^*t*−1^} denotes the known network states.

Formally, most policies can be represented as decision trees. We use *Q*_*i*_ to denote the possible policy space for agent *r*_*i*_, and *Q*_−*i*_ denotes the sets of policy trees for all agents except *r*_*i*_. With a programming approach, it is required that we generate incrementally the sets of useful policies for each agent. Thus, a joint policy Π = ×_*i* ∈ *N*_{*π*_*i*_} is a vector of policy trees. Evaluating a joint policy can then use the following formulation:

$$\begin{array}{c} \begin{array}{c} V\left(s,\Pi \right)=\sum_{\omega \in \Omega }P\left(\omega |s,\Pi \right)\left[\sum_{s^{\prime }\in S}P\left(s^{\prime }|s,\pi ,\omega \right)V\left(s^{\prime },\Pi \left(\omega \right)\right)\right] \end{array} \end{array}$$ (1)

where Π(*ω*) is the joint policy of subtree selected after observation *ω*. So we get the utility function as:

$$\begin{array}{c} \begin{array}{c} U\left(b_{i},\Pi_{i}\right)=\sum_{s\in S}\sum_{\Pi_{-i}\in Q_{-i}}b_{i}\left(s,\Pi_{-i}\right)V\left[s,\left\{\Pi_{-i},\pi_{i}\right\}\right] \end{array} \end{array}$$ (2)

Therefore, the essence of this framework is to find a set of *n* policies to maximize a total reward function from finite horizon *T* under initial belief state *p*_0_, and the expected joint reward is given by $E(\sum_{t=0}^{T}R\left(s^{t},\Lambda^{t}\right)|p_{0})$.

## A Resource-aware Approach for Multi-agent Multi-channel Access

In this section, we demonstrate an agent’s decision-making process based on current observation and resource perception, and analyze the computational complexity under the instincts of *no-information-sharing*.

### Resource Awareness Policy Generation

From the idealistic view of the *Shannon’s* theory [22], the optimal available resources under an ideal state for *r*_*i*_ is:

$$\begin{array}{c} \begin{array}{c} Cap_{i}=B_{n}\sum_{j=1}^{N-1}\left\{\left(1-p_{m}\right)log_{2}\left(1+\frac{g_{i}^{2}p_{j}^{o}}{\sigma_{j}^{o}}\right)+p_{m}log_{2}\left(1+\frac{g_{i}^{2}p_{j}^{o}}{\sigma_{i}^{p}+g_{i}^{2}p_{i}^{p}}\right)\right\} \end{array} \end{array}$$ (3)

where *B*_*n*_ is the channel bandwidth and *p*_*m*_ is the channel state misperception probability. $\sigma_{j}^{o}$ and $\sigma_{i}^{p}$ respectively denote the noise variances from other agents and *r*_*i*_ affected channel *ch*_*i*_. $p_{j}^{o}$ and $p_{i}^{p}$ respectively denote the communication power of other agents and *r*_*i*_. *g*_*i*_ is the channel sensing gain. However, in the presence of sensing error, not only the sensing and access policy but also the operating characteristics of the channel sensor affect the performance of the network and the interference perceived by all the agents. The loss of resources caused by interferences are:

$$\begin{array}{c} \begin{array}{c} △Cap_{i}=B_{n}\sum_{j=1}^{N-1}[log_{2}\left(1+\frac{g_{i}^{2}p_{j}^{o}}{\sigma_{j}^{o}}\right)-log_{2}\left(1+\frac{g_{i}^{2}p_{j}^{o}}{\sigma_{i}^{p}+g_{i}^{2}p_{i}^{p}}\right)]p_{m} \end{array} \end{array}$$ (4)

As a result, agent *r*_*i*_ can obtain the idealistic expectation channel resources in *C*_2_ as:

$$\begin{array}{c} \begin{array}{c} ECap_{i}=\sum_{i=1}^{|C_{2}|}\left(Cap_{i}-△Cap_{i}\right) \end{array} \end{array}$$ (5)

We can see that the agent can access the network interval sequence independently, and this follows the same negative exponential distribution *G*(*t*) = 1−*e*^−*μ*_*i*_*t*^, where *μ*_*i*_ is the channel free probability. Thus, we can get the probability of agent *r*_*i*_ to choose and access channel *c*_*j*_ as:

$$\begin{array}{c} \begin{array}{c} p_{i,j}^{p}=p_{i,j}^{s}V\left(ECap_{i},\Pi_{i}\right)log_{2}\left(1+\frac{\nu_{i,j}}{ln\frac{0.2}{BER_{i,n}}}\right) \end{array} \end{array}$$ (6)

We can use $p_{i,j}^{p}$ and $p_{i,j}^{s}$ to denote the probability that agent *r*_*i*_ select channel *c*_*j*_ and the probability of channel *c*_*j*_ being sensed idle respectively. $\nu_{i,j}=\frac{ECap_{j}\times p_{j}}{E(△Cap_{j})+N_{0}}$ is the Signal to Interference plus Noise Ratio (SINR) for agent *r*_*i*_ from the other agents in channel *c*_*j*_. This problem cannot be solved in one stage, and as such, should be done in an iterative manner. Therefore, based on the above analysis, we use Eqs (5) and (6) to obtain the policy tree and a target BER equal to $BER_{i,n}\approx \sigma_{1}exp\left[\frac{-\sigma_{2}\zeta_{i,n}}{2^{b_{i,n}}-1}\right]$, where *σ*_1_ and *σ*_2_ are Lagrangian multipliers, *b*_*i*, *n*_ is the number of bits per symbol in channel *c*_*n*_, and *ζ*_*i*, *n*_ is the Signal to Noise Ratio (SNR) for the receiver agent *r*_*i*_ in channel *c*_*n*_. Consequently, we adopt the utility function design in [23]:

$$\begin{array}{c} \begin{array}{c} U\left(b,\pi \right)=\mu_{1}\sum_{c_{i}\in C_{2}}p_{i,j}^{p}log\left(\mu_{2}c_{i}^{h}k_{i}\right)-cost_{i} \end{array} \end{array}$$ (7)

where the product $c_{i}^{h}k_{i}$ is the bandwidth (i.e. transmission rate), $c_{i}^{h}$ is the size of access channel in Hz, *k*_*i*_ is the spectral efficiency^2^ in bits per symbol per Hz due to adaptive modulation, and *μ*_1_ and *μ*_2_ are constants that depend on the communications protocol and agent communication system performance, respectively. *cost*_*i*_ is the communication consumption, which relates to the agent’s hardware system. The optimal policy is therefore π* = *argmax*[*U*(*b*, *π*_*i*_)].

### Dynamic Local Search

Based on the model described above, agent *r*_*i*_ cannot get a full view of the state of the system, since it can only use its observation to update its actions. The goal of this problem’s model is to come up with a joint policy Π* = $\times_{1\leq i\leq N}\left\{\pi_{i}^{t}\right\}$, which can maximize the expected reward of all the agents over a finite horizon. The belief space is a sufficient statistic [21], and can be independent of the decision time. We remark that *r*_*i*_ can only infer what action its neighbors may take, but the inference or conflict is inevitable. At each time slot, we can compute the expected value of a policy as follows:

$$\begin{array}{c} \begin{array}{c} E\left(V_{\pi }^{t}\left(\Omega^{t-1},\omega^{t}\right)\right)=R\left(\Omega^{t-1},\left\langle \omega^{t},\pi^{t}\right\rangle \right)+\sum_{S^{\prime }\in S}P\left(\Omega^{t-1},\left\langle \omega^{t},\pi^{t}\right\rangle ,S^{\prime }\right)\\ \cdot \sum_{\omega \in \Omega }O\left(S^{\prime },\left\langle \omega^{t},\pi^{t}\right\rangle ,\omega^{\prime }\right)\cdot V_{\pi }^{t+1}\left(S^{\prime },\omega^{\prime }\right) \end{array} \end{array}$$ (8)

Solutions to a finite-horizon POMDP can be represented as a decision tree, where nodes denote the actions and arcs denote the observations. Similarly, solving a finite-horizon Dec-POMDP with known state space can be formulated as a multiple vector of horizon *T* policy tree searching process.

**Algorithm 1:** Resource aware policy search for agent *r*_*i*_.

**Require:**

 Set *g*_0_ = 0; ℜ_0_ = {*Φ*}; ℜ ∈ *Q*_*i*_;

**Ensure:**

$\exists \;\Pi^{*}=\times_{i\in N}\pi^{*}\;and\;\forall \;v\left(\pi_{i}\right)\leq v\left(\pi_{i}^{*}\right)$;

1: **for** *each* *r*_*i*_ **do**

2:  *random* *select* *ploicy* *candidates* *set* {*η*_*i*_}*from* ℜ;

3:  *g*_*i*_(*t*) = $\max_{k\in (i⋃N_{i})}g_{k}$;

4:  **for** *all* *π*_*i*_ ∈ *η*_*i*_ **do**

5:   *excute* *π*_*i*_ *to* *obtain* *ω*_*t*_;

6:   *compute* *g*_*i*_(*t*);

7:   **if** $g_{i}(t)>\hat{V}(\pi_{0})$**then**

8:    π* = *π*_*i*_;

9:   **else**

10:    *prune π_i_ and get new* $\pi_{i}^{\prime }$

11:   **end if**

12:   $\eta_{i}^{\prime }$ = *argmax* {Π′ ∈ ℜ|*V*(η′) > *R*(*η*)};

13:  **end for**

14:  *return* π* → Π*;

15: **end for**

As shown in Algorithm 1, ℜ ∈ *Q*_*i*_ denotes the random initialized policy space with completely unspecified candidate policies. *g*_*i*_ = $EV(\pi_{i}^{*})$ − $V(\pi_{i}^{t})$ is the difference in value between the expected policy and the current one. In the beginning of each searching round, randomly select *η*_0_ from ℜ, if *g*_*i*_’s value is bigger than $\hat{V}\left(\pi_{0}\right)$, then map *π*_*i*_ to $\pi_{i}^{*}$. If not, prune the inappropriate *π*_*i*_ and search new $\pi_{i}^{\prime }$. We assume that the partial policy with the highest heuristic value is selected, and the provided value of $\hat{V}\left(\pi_{0}\right)$ is the *lower bound* for an optimal joint policy, which can be used to prune the search space. If *r*_*i*_ has the minimum *g*_*i*_ value in one round, then it will get priority to access its {*C*_2_}. Other agents are constantly updated to the new strategy, and after finite times evaluation and exploration that they can get all the apposite policies to fix the *g*_*i*_ value. In a limited belief space, by retrieving the limited policies space, and the state transition probability approaching the optimal values, similarly, the decision can approach the optimal policy $\Pi_{i}^{*}$. At each time slot, the computation of *g*_*i*_ performs a summation over all possible network states and observations, and so the time complexity of this algorithm is *O*((|*S*|⋅|*Q*_*i*_|)^*T*^). The value of a policy is highly dependent on the other agents’ beliefs and the current system status, whereas, without sharing, the policy regeneration can only be derived on the basis of the reckoned joint policies. We define the policy update function as:

$$\begin{array}{c} \begin{array}{c} \left\{\begin{array}{c} \pi_{i}^{\prime }=argmax\Xi_{\Pi }\left[R\left(\pi_{i}^{\prime }|\delta_{i}^{t},V\left(\pi^{t}\right)\right)\right]\\ V\left(\pi^{t}\right)=\frac{1}{|C_{2}|}\sum_{1}^{|C_{2}|}R\left(ECap_{i}|a^{t},\pi^{t},\delta_{i}^{t}\right); \end{array}\right. \end{array} \end{array}$$ (9)

where *Ξ*_*π*_ represents the conditional expectation given that policy *π*_*i*_ is employed, and *B*_0_ is the initial belief, which can be the stationary distribution of the network state. $\delta_{i}^{t}$ is the knowledge, consisting of two parts: channels observation *ω*^*t*^ and the known status $\Omega_{i}^{t}$. The search strategy performs a summation over all possible network states from agents’ observations. Since each policy specifies different actions over possible histories of observations, the number of possible policies for agent *r*_*i*_ is $O(|A_{i}{|}^{\frac{{\left|\Omega \right|}^{T}-1}{|Q_{i}|-1}})$. In consequence, the time complexity of finding the optimal policy by searching this space is: $O(|A_{i}{|}^{\frac{{\left|\Omega \right|}^{T}-1}{|Q_{i}|-1}}\cdot |S|\cdot |Q_{i}{\left|\right)}^{T})$.

## A Decision Theoretical Approach for Multi-channel Access

In the previous sections, we proposed a random searching solution without coordination. This method has very high computational complexity and time cost. But from a practical point of view, each agent can be aware of its neighbor. Therefore, with the neighbor’s policy sharing, the agent *r*_*i*_ can get a proximate full local observation. Consequently, we refer to the design in [21], and the multi-agent finite horizon Dec-POMDP model can decompose into several single-agent POMDP decision problems.

### Neighbor-Aware Policy Generation

In order to solve a single-agent POMDP, we introduce neighbor policies ${\bar{\pi }}_{i}$ as a new parameter to the knowledge *δ*_*i*_, and the joint policy of *n* neighbors is formulated as ${\bar{\Pi }}_{i}=\times_{i\in n}\left\{{\bar{\pi }}_{i}\right\}$. Therefore, we augment the state space to be $ℑ=\{S\times \bar{S}\}$ , where the second set $\bar{S}$ is the state variables of the other agents’ beliefs. In consequence, we resolve and upgrade the Dec-POMDP to a POMDP model as a tuple $<ℑ,A,T,\Omega ,O,R,\{\delta_{i}\}>$. All variable definitions remain unchanged, and to accomplish this, we factor the transition distribution into two terms: $T[(s^{\prime },\bar{s^{\prime }})|a,{\bar{\Pi }}_{i}(\bar{s}),(s,\bar{s})]=T[s^{\prime }|a,{\bar{\Pi }}_{i}(\bar{s}),\bar{T}(\bar{s^{\prime }}|s^{\prime },a,{\bar{\Pi }}_{i}(\bar{s})]$ , and the *upper bound* of the POMDP value function can be reached through the complete observation. In consequence, the belief update function can be denoted as:

$$\begin{array}{c} \begin{array}{c} b\left(s^{\prime }\right)=P\left(s^{\prime }|\omega ,a,b\right)=\frac{O\left(s^{\prime },a,\omega \right)\sum_{s\in ℑ}T\left(s,a,s^{\prime }\right)b\left(s\right)}{P(\omega |a,b)} \end{array} \end{array}$$ (10)

The value function of a POMDP is defined over the space of beliefs, where a belief state *b* represents a probability distribution over states. The optimal value of policy π* can then be approximated as:

$$\begin{array}{c} \begin{array}{c} V_{\pi^{*}}\left(b\right)=\max_{a\in A}\left\{R_{\delta }\left(b,a\right)+\lambda \sum_{\omega \in \Omega }p\left(\omega |b,a\right)\cdot V^{*}\left(b,a,\omega \right)\right\} \end{array} \end{array}$$ (11)

### Heuristic Local Policy Search

Modeling our problem as a POMDP model is to search for the optimal policy π*, and maximize the expected reward over a finite horizon-*T* policy distribution over states. Formally, a belief state *b*_*t*+1_ = *P*(*s*_*t*+1_|*δ*^*t*^, *δ*^*t*−1^, …., *δ*^0^) is a probability distribution over states conditioned on knowledge $\delta_{i}^{t}$. In order to avoid a heuristic with unbounded input (the knowledge can be arbitrary), a traditional approach is to learn a mapping from belief states to actions, which is from the known knowledge $\delta_{i}^{t}$. But in discrete worlds, beliefs can only be represented by a state with probabilities. We represent the regeneration process of belief states by sampling. A sample *x* is annotated with a numerical importance factor to account for the difference in the sampling distribution.

Heuristic search is based on the decomposition of the evaluation function into a sequence of exact sub-evaluations. As aforementioned, we denote *q*^*t*^ as an arbitrary depth *t* policy vector extract from policy vector *Q*^*T*^, and {*q*^*t*^, *Q*^*T*−*t*^} constitutes a complete policy vector of depth *T*. This allows us to decompose the policy vector into any *t* depth vector, and the value of the completion is:

$$\begin{array}{c} \begin{array}{c} V\left(Q^{T-t}|\left\{q^{t},p_{0}\right\}\right)=V\left(p_{0},q^{t}\right)+H^{T-t}\left(Q^{T-t}|q^{t},p_{0}\right) \end{array} \end{array}$$ (12)

where *H*(*q*) is the heuristic function, and the value of *Q*^*T*−*t*^ depends on the previous execution and the underlying state distribution at time *t*. In consequence, we can describe the heuristic function as:

$$\begin{array}{c} \begin{array}{c} H^{T-t}\left(Q^{T-t}\right)=\sum_{s\in S}P\left(s|p_{0},q^{t}\right)H^{T-t}\left(s\right) \end{array} \end{array}$$ (13)

As in Algorithm 2, randomly extract a sample *q*^*t*^ from the possible policy space *Q*, and each node in the tree is a belief state *b*_*i*_. For each encountered state *x*_*i*_, belief state *b*_*i*_ is updated to include the new state $x_{i}^{\prime }$. In each sample searching, the agent selects the policy *b*′ at the greatest value. The sampling path terminates when it reaches a sufficient depth of the bounds of *T*_*q*_, and goes back to the root so as to improve the *upper* and *lower bound* estimates. The search moves towards π* only with the acceptance probability *P*(*b*^0^), otherwise it remains at *b*′. At this point, the node *b*^0^ becomes the root of the new search tree, and the remainder of the tree is pruned, as all other beliefs are now impossible. The search in new sample trees would not stop until there appears a policy to meet the resource requirements. Obviously, under a statistical hypothesis, the searching process converges to the expected distribution at a rate of $\frac{1}{\sqrt{H}}$, and *H* denotes the sample size.

Algorithm 2: Sample extract-based search for agent *r*_*i*_.

**Require:**

 *random extract sample* $\left\{q_{i}^{t}\right\}$ *from Q*; *v*(*b*_0_) = 0;

**Ensure:**

  $\exists \;\forall \;v\left(\pi_{i}\right)\leq v\left(\pi_{i}^{*}\right)$;

1: *random extract sample* $\left\{q_{i}^{t}\right\}$ *from Q*;

2: **for** *each* $q_{i}^{t}$ **do**

3:  *qualify* *T*_*q*_;

4:  **repeat**

5:   **for** *each state x_i_ from b_i_* **do**

6:    *compute* $b\left(x_{i}^{\prime }\right),\;x_{i}^{\prime }\leftarrow T\left(x_{i},a,x_{i}^{\prime }\right);$

7:    **if** *b*′ ∈ *q*^*t*^ **then**

8:     *continue* *to* *next* *b*_*i*_;

9:    **else**

10:     *add* *b*′ *to* *T*_*q*_;

11:     **if** *U*(*b*)<*U*(*b*′) **then**

12:      *b*^0^ = *b*′;

13:     **end if**

14:    **end if**

15:    *prune* *q*^*t*^ *other* *than* *b*^0^;

16:   **end for**

17:   *generate* *new* *q*^*t*^′ *from* *root* *b*^0^;

18:  **until** $P\left(b^{0}\right)=min\left(1,\frac{b\left(x^{\prime }\right)T\left(x^{\prime }|a,x\right)}{\sum_{x\in b}b\left(x\right)T\left(x^{\prime }|a,x\right)}\right);$

19: **end for**

20: *return* π*;

A concise example is described in the following to illustrate our algorithmic process. We demonstrate a minimize-scale: two agents coordination. For each agent *r*_*i*_, its action space has two actions {*Listen*, *Switch*}. These actions achieve channel perception, switch to other channels or stay in current channel, respectively. Each agent can sense two channels and choose one to access. The coordination has two states: establish a connection (R) or fail (W), denoted by *S* = <*R*, *W*>. The channel state misread probability is 0.3. The action-state transfer probability table as in Table 1, the initial joint action is <*S*, *S*>.

**Table 1:** State-action transfer probability.

Action	S,S	L,S	S,L	L,L
state				
W	0.09	0.21	0.21	0.49
R	0.49	0.21	0.21	0.09

**Fig 2:**
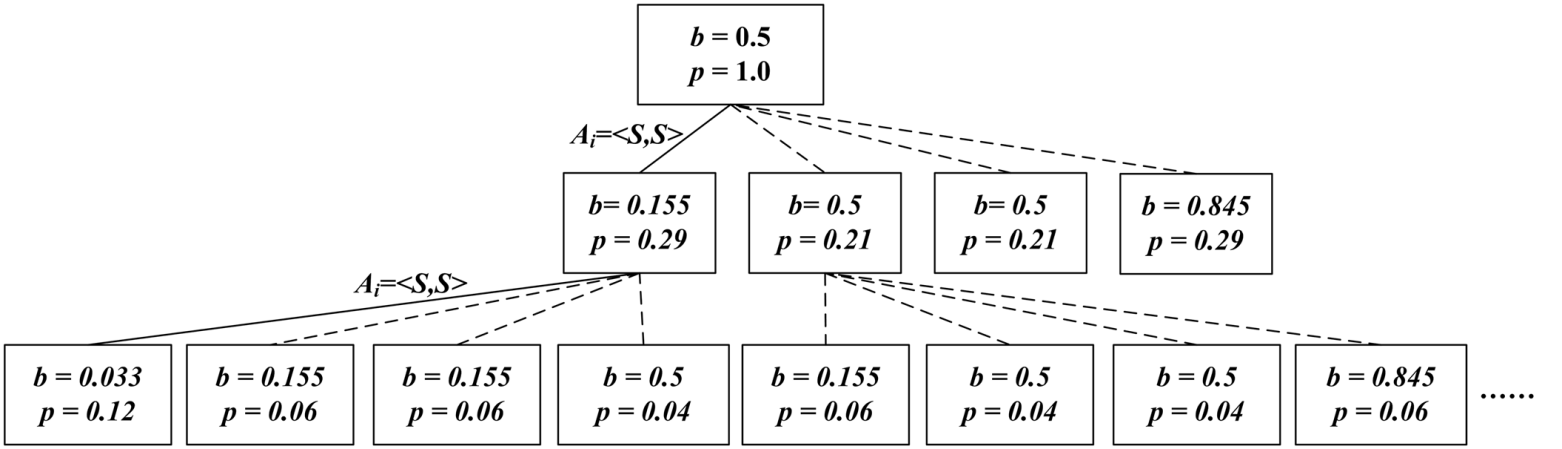
Beliefs Update Processing. A 3-step policy tree captured from Table 1, each of which can be conditioned on the outcome of previous actions. Each node is labeled with the action that should be taken if it is reached.

We define the highest reward (+50) to be the case when both agents get a good resource acquisition. A lower reward (-20) is agents’ access in two different channels, and they can connect but with low resource acquisition. The worst case is lose connection (-100), and the cost of *Listen* is (-10). As shown in Fig 2, both agents start out with an initial belief state of b(s) = 0.5, and the discount factor is *γ* = 0.9. The first joint action at this belief is < *Listen*, *Listen* >, the reward is (-20). As such, each agent has its own observation and network belief. In order to get a better reward, each agent removes all of the joint beliefs that are not consistent with its entire observation. After policies sharing, there is only a single possible belief *b*(*W*) = 0.033, and the optimal joint action for this belief is < *Switch*, *Switch* >.

It should be noted that there exists hidden competitions between agents for the finite resources (each agent wants to get more resources), that is, there should exist optimal joint policies to reach the Pareto optimal. But, it is infeasible for Dec-POMDP model because of the partial observation that we briefly described in the previous sections. In our design, it can finally reach the approximate Pareto optimal after a finite search. Therefore, it means that, in the finite belief space, there exists a pair of policies *π* = (*π*_1_, *π*_2_) such that: $\forall_{\pi_{1}^{\prime }}\left(V_{1}\left(\pi_{1},\pi_{2}\right)\right)\geq V_{1}\left(\pi_{1}^{\prime },\pi_{2}\right)⋀\forall_{\pi_{2}^{\prime }}\left(V_{2}\left(\pi_{1},\pi_{2}\right)\right)\geq V_{2}\left(\pi_{1},\pi_{2}^{\prime }\right)$. That is, for each agent, playing *π*_*i*_ gives an equal or higher expected resource than playing $\pi_{i}^{\prime }$. So both policies are best responses to each other.

## Implicit Competition Modeling and Equilibrium Analysis

As aforementioned, there exists hidden competition between agents for the finite channel resources, and techniques for eliminating dominated strategies in solving a POMDP are very closely related to techniques for eliminating dominated strategies in solving games in normal form [24]. From the game perspective, agents can get their locally optimal policy according to the Best Response (BR) dynamic iteration. In a general game, each agent negotiates and chooses the channels to maximize its payoff based on the channel situation in the last time slot observation, but the other agents (interference) can not change their channels simultaneously. However, BR does not guarantee convergence in all cases, and the stable state can not always be with the optimal overall reward. Hence, we study the characteristics of the multi-channel access game and its sub-optimal as in the following.

### Implicit Competition Game Model

According to the aforementioned, the access problem can be defined as a cooperative game $G=\langle R,S,D_{i},R\rangle$, where the definitions of R and *S* are unchanged, *D*_*i*_ = ×_1 ≤ *i* ≤ *k*_{*π*_*i*_} is the finite set of policies available to agent *r*_*i*_, R denotes the reward. We use *θ*_*i*_(*π*_*i*_) to denote the probability distribution assignment over policies available to agent *r*_*i*_. Since agents select their policies simultaneously, agent *r*_*i*_’s belief about the other agents’ likely policies can be denoted as *θ*_−*i*_. If we define *V*_*π*_*i*__(*s*, *θ*_−*i*_) = ∑_*d*_−*i*__ *θ*_−*i*_(*d*_−*i*_)*V*_*i*_(*π*_*i*_, *d*_−*i*_), then $B_{i}\left(\theta_{-i}\right)=\left\{\pi_{i}\in D_{i}|V_{i}\left(\pi_{i},\theta_{v}\right)\geq V_{i}\left(\pi_{i}^{\prime },\theta_{-i}\right)\right\}$ denotes the best response function of agent *r*_*i*_, which is the set of policies for agent *r*_*i*_ that maximize its value of some belief about the policies of the other agents *d*_−*i*_. Any policy that is not a best response to some belief can be abandoned.

Algorithm 3: General framework of competition equilibrium.

**Require:**

 ∃ *θ*_−*i*_ = {*b*_1_, …, *b*_*i*−1_, *b*_*i*+1_, …};

**Ensure:**

 ∀ *s* ∈ *S* and $v\left(\pi_{i}\right)\leq v\left(\pi_{i}^{*}\right)$;

1: **for** *each* *episode* **do**

2:  *Initialize* *get* *state* *S* *and* *D*;

3:  **repeat**

4:   $compute\;V_{\pi_{i},d_{-i}}\left(b_{i}\right)\leftarrow O\left(s_{i},a,s_{i}^{\prime }\right);$

5:   **if** $V_{\pi_{i},d_{-i}}\left(b_{i}\right)<R_{min}^{i}$ **then**

6:    $prune\;\pi_{i}\;and\;get\;new\;\pi_{i}^{\prime };$

7:   **else**

8:    *return* *π*_*i*_ *to* *D*′;

9:   **end if**

10:  **until** $U_{i}\left(b_{i}\right)=\max\sum_{\pi_{i}\in D^{\prime }}b_{i}\left(s,d_{-i}\right)V_{\pi_{i}}\left(s,\theta_{-i}\right);$

11: **end for**

12: *return* *π*_*i*_ → π*;

As in Algorithm 3, in a cooperative game, the reward functions for the game correspond to the reward functions of the POMDP, and an agent’s belief is a distribution synthesization over the possible policies of the other agents. For each agent *r*_*i*_, a belief is defined as a distribution over *S*×*D*_−*i*_, where the distribution is still denoted by *b*_*i*_, and the utility of *b*_*i*_ is:

$$\begin{array}{c} \begin{array}{c} U_{i}\left(b_{i}\right)=\max\sum_{\pi_{i}\in D^{\prime }}b_{i}\left(s,d_{-i}\right)V_{\pi_{i}}\left(s,\theta_{-i}\right) \end{array} \end{array}$$ (14)

Given the set of policies and the reward function for a horizon-*t*’s game, the sets of policies and value functions for the *t* horizon game are constructed by exhaustive backup. When a horizon-*t*’s POMDP is represented in the normal form with implicit competition, the policy sets include all depth-*t* policy trees. Each policy profile is associated with a belief vector B, reresenting the expected *t*-step cumulative reward achieved for each potential start state by following an apposite joint policy, whiles the size of the policy set for each agent *r*_*i*_ is more than $A_{i}^{{\left|O\right|}^{t}}$, which is doubly exponential in the horizon-*t*. Because of the large sizes of the candidate policy sets, it is usually not feasible working directly. The search algorithm (Algorithm 2) we present in this paper only partially alleviates this problem by performing iterative elimination of dominated policies at each stage in the construction of the normal form representation, rather than waiting until the construction completes. Considering an *N*-player implicit competition game, we can formulate the game subject as:

$$\begin{array}{c} \begin{array}{c} \left\{\begin{array}{c} \sum_{n=1}^{K}\omega ln\left(1+\frac{V\left(b_{i,n}\right)G_{i,i,n}\sigma_{3}}{\delta_{i}^{2}+\sum_{j=1}^{K}p_{j,n}g_{j,i,n}}\right)-R_{min}^{i}\geq 0\\ \sum_{n=1}^{K}\omega ln\left(1+\frac{V\left(b_{i,n}\right)G_{i,i,n}\sigma_{3}}{\delta_{i}^{2}+\sum_{j=1,j\neq i}^{K}p_{j,n}g_{j,i,n}}\right)-R_{exp}^{i}\geq 0 \end{array}\right. \end{array} \end{array}$$ (15)

In this constraints equations, *r*_*i*_’s desired reward is no less than $R_{min}^{i}$, and this guarantees a minimum level of resources achieved by each agent. *v*(*b*_*i*, *n*_) is value of the belief distribution of agent *r*_*i*_’s access in channel *c*_*n*_, $\delta_{i}^{2}$ is the variance of the white Gaussian noise, and $\sigma_{3}=\frac{\sigma_{2}}{ln\sigma_{1}}$. $R_{exp}^{i}$ is the expected resource reward, and *G*_*i*, *j*, *n*_ is the channel gain between two agents in channel *c*_*n*_, and all policies should meet $\sum_{i=1}^{K}v\left(b_{i,n}\right)\leq R_{\pi^{*}}\left(b,K\right)$. The existence and stability of the competition will be investigated in the following subsection.

### Evolutionary Equilibrium Analysis with Replicator Dynamics

In a multi-agent multi-channel access game, the stable state can be defined as the following: a joint policy Π* is and only if, for each agent and an arbitrary policy *π* in its policy space, *v*_*i*_(π*) ≥ *v*_*i*_(*π*, *θ*_−*i*_) is always satisfied. Consequently, the process of this game can be modeled as a replicator dynamics, and this can be derived for each agent separately.

We consider a concise example with two new access agents *r*_1_ and *r*_2_. These agents appear first in the network with some spared channel opportunity (i.e., *c*_1_ to *c*_*n*_). With this specification, we analyze the evolutionary equilibrium for both deterministic and stochastic models. For the hidden competition among agents, the evolutionary equilibrium can be obtained as Replicator Dynamics solution [25], where *χ*_*i*_ denotes the proportion of the eager channel resources that agents can get, and the replicator dynamics can be defined as the following:

$$\begin{array}{c} \begin{array}{cc} \frac{\partial \chi_{i}^{b_{i}}\left(t\right)}{\partial t}= & \sigma \chi_{i}^{b_{i}}\left(t\right)\left[v_{i}^{b_{i}}-{\bar{v}}^{b_{i}}\right]\\ = & \sigma \chi_{i}^{b_{i}}\left(t\right)\left[U\left(\pi ,\Pi_{-i}\right)-\bar{U}\left(\Pi_{-i}\right)\right] \end{array} \end{array}$$ (16)

where ${\bar{v}}^{\left(c_{i}\right)}$ is the estimated average reward for other agents in channel *c*_*i*_, and the function *U* is defined in Eq (7). With the two agents case, the evolutionary equilibrium is obtained as the solution of the following equation:

$$\begin{array}{c} \begin{array}{cc} \mu_{1}log & \left(\mu_{2}\frac{\chi_{1}^{b_{1}}U\left(b_{1}\right)}{\chi_{1}^{b_{1}}U\left(b_{1}\right)+\left(1-\chi_{2}^{b_{2}}U\left(b_{2}\right)\right)}\right)-v_{\pi_{1}}\left(b_{1}\right)\\ & =\mu_{1}log\left(\mu_{2}\frac{\chi_{2}^{b_{2}}U\left(b_{2}\right)}{\left(1-\chi_{1}^{b_{1}}U\left(b_{1}\right)\right)+\chi_{2}^{b_{2}}U\left(b_{2}\right)}\right)-v_{\pi_{2}}\left(b_{2}\right) \end{array} \end{array}$$ (17)

where the terms on both sides of the equation are the rewards that the new access agents can get from their beliefs *b*_1_ and *b*_2_, respectively. Accordingly, the stability of the evolutionary equilibrium can be analyzed using the following Jacobian matrix:

$$\begin{array}{c} \left[\begin{array}{c} \frac{\partial \sigma \chi_{1}^{b_{1}}\left[U\left(\pi ,\Pi_{-1}\right)-\bar{U}\left(\Pi_{-1}\right)\right]}{\partial \chi_{i}^{b_{1}}}\frac{\partial \sigma \chi_{1}^{b_{1}}\left[U\left(\pi ,\Pi_{-1}\right)-\bar{U}\left(\Pi_{-1}\right)\right]}{\partial \chi_{i}^{b_{2}}}\\ \frac{\partial \sigma \chi_{1}^{b_{2}}\left[U\left(\pi ,\Pi_{-2}\right)-\bar{U}\left(\Pi_{-2}\right)\right]}{\partial \chi_{i}^{b_{1}}}\frac{\partial \sigma \chi_{1}^{b_{2}}\left[U\left(\pi ,\Pi_{-2}\right)-\bar{U}\left(\Pi_{-2}\right)\right]}{\partial \chi_{i}^{b_{2}}} \end{array}\right]=\left[\begin{array}{c} J_{1,1}J_{1,2}\\ J_{2,1}J_{2,2} \end{array}\right] \end{array}$$ (18)

where

$$\begin{array}{c} \begin{array}{cc} J_{1,1}= & \sigma \{Z_{2}-v_{\pi_{1}}\left(b_{1}\right)-\chi_{1}^{b_{1}}\left(Z_{2}-v_{\pi_{1}}\left(b_{1}\right)\right)-\left(1-\chi_{1}^{b_{1}}\right)\times \left[\mu_{1}p_{1,2}^{p}log\frac{\mu_{2}c_{1}^{h}k_{1}}{Z_{1}}-v_{\pi_{2}}\left(b_{2}\right)\right]\}\\ & -\sigma \chi_{1}^{b_{1}}\{\frac{\mu_{1}U\left(b_{1}\right)}{\chi_{1}^{b_{1}}U\left(b_{1}\right)+\left(1-\chi_{2}^{b_{2}}U\left(b_{2}\right)\right)}+Z_{2}-v_{\pi_{1}}\left(b_{1}\right)\\ & -\frac{\mu_{1}\chi_{1}^{b_{1}}U\left(b_{1}\right)}{\chi_{1}^{b_{1}}U\left(b_{1}\right)+\left(1-\chi_{2}^{b_{2}}U\left(b_{2}\right)\right)}-\mu_{1}p_{1,2}^{p}log\frac{\mu_{2}c_{2}^{h}k_{2}}{Z_{1}}+v_{\pi_{2}}\left(b_{2}\right)+\frac{1-\chi_{1}^{b_{1}}\mu_{1}U\left(b_{1}\right)}{Z_{1}}\} \end{array} \end{array}$$ (19)

$$\begin{array}{c} \begin{array}{cc} J_{1,2}= & \sigma \chi_{1}^{b_{1}}\{-\frac{\mu_{1}U\left(b_{1}\right)}{\chi_{1}^{b_{1}}U\left(b_{1}\right)+\left(1-\chi_{2}^{b_{2}}U\left(b_{2}\right)\right)}-\frac{\left(1-\chi_{1}^{b_{1}}\right)\mu_{1}U\left(b_{1}\right)}{Z_{1}}\\ & +\frac{\mu_{1}\chi_{1}^{b_{1}}U\left(b_{1}\right)}{\chi_{1}^{b_{1}}U\left(b_{1}\right)+\left(1-\chi_{2}^{b_{2}}U\left(b_{2}\right)\right)}\} \end{array} \end{array}$$ (20)

$$\begin{array}{c} \begin{array}{cc} J_{2,1}= & \sigma \chi_{1}^{b_{1}}\{-\frac{\mu_{1}U\left(b_{1}\right)}{\chi_{1}^{b_{1}}U\left(b_{1}\right)+\left(1-\chi_{2}^{b_{2}}U\left(b_{2}\right)\right)}-\frac{\left(1-\chi_{2}^{b_{2}}\right)\mu_{1}U\left(b_{1}\right)}{Z_{1}}\\ & +\frac{\mu_{1}\chi_{1}^{b_{1}}U\left(b_{1}\right)}{\chi_{1}^{b_{1}}U\left(b_{1}\right)+\left(1-\chi_{2}^{b_{2}}U\left(b_{2}\right)\right)}\} \end{array} \end{array}$$ (21)

$$\begin{array}{c} \begin{array}{cc} J_{2,2}= & \sigma \{Z_{2}-v_{\pi_{1}}\left(b_{1}\right)-\chi_{1}^{b_{1}}\left(Z_{2}-v_{\pi_{1}}\left(b_{1}\right)\right)-\left(1-\chi_{1}^{b_{1}}\right)\times \left[\mu_{1}p_{1,2}^{p}log\frac{\mu_{2}c_{1}^{h}k_{1}}{Z_{1}}-v_{\pi_{2}}\left(b_{2}\right)\right]\}\\ & -\sigma \chi_{2}^{b_{2}}\{\frac{\mu_{1}U\left(b_{2}\right)}{\chi_{1}^{b_{1}}U\left(b_{1}\right)+\left(1-\chi_{2}^{b_{2}}U\left(b_{2}\right)\right)}+Z_{2}-v_{\pi_{1}}\left(b_{1}\right)\\ & -\frac{\mu_{1}\chi_{2}^{b_{2}}U\left(b_{2}\right)}{\chi_{1}^{b_{1}}U\left(b_{1}\right)+\left(1-\chi_{2}^{b_{2}}U\left(b_{2}\right)\right)}-\mu_{1}p_{1,2}^{p}log\frac{\mu_{2}c_{2}^{h}k_{2}}{Z_{1}}+v_{\pi_{2}}\left(b_{2}\right)+\frac{1-\chi_{2}^{b_{2}}\mu_{1}U\left(b_{2}\right)}{Z_{1}}\} \end{array} \end{array}$$ (22)

where *Z*_*i*_ specified as $Z_{1}=\left(1-\chi_{1}^{b_{1}}\right)U\left(b_{1}\right)+\left(1-\chi_{2}^{b_{2}}\right)U\left(b_{2}\right)$ and $Z_{2}=\mu_{1}log\frac{\mu_{2}c_{1}^{h}k_{1}}{\chi_{1}^{b_{1}}U\left(b_{1}\right)+\chi_{2}^{b_{2}}U\left(b_{2}\right)}$. The two eigenvalues of J can be obtained from $\Delta \left(J\right)=\frac{J_{1,1}+J_{2,2}\pm \sqrt{4J_{1,2}J_{2,1}+{\left(J_{1,1}-J_{2,2}\right)}^{2}}}{2}$, and the evolutionary equilibrium is stable if these two eigenvalues have negative real parts [23].

### Approximate Fair Maximization Policy Analysis

Among the different Cooperative Game solutions, it is important to note that the issue about fairness in this context, e.g., new access agents, is different from the case of resource occupation among the early-existing agents in the network. In this section we will analyze approximate fairness of the game, and discuss the feasibility of the proposed neighbor-aware channel access scenario in the previous section. In this scenario, the approximate Pareto optimal result can satisfy all agents’ minimum requirements. Typically, if a channel is occupied, the other agents should be denied access to the frequency band.

In the proposed competitive game model, a virtual-feasible resource access assignment set is existent, hence, we can use a bounded set F = ${\left\{{℧}_{min}^{1},{℧}_{min}^{2},...,{℧}_{min}^{C_{2}}\right\}}^{T}$, which denotes the minimum resource required of the game. The vector *r* = ${\left\{R_{exp}^{1},R_{exp}^{2},...,R_{exp}^{C_{2}}\right\}}^{T}$ represents the set of rewards for the access agents. The reward vector $r\in R^{K+1}$ in the *K* channels can form the fairness problem ϕ(F,r). It has been shown that there exists a unique equilibrium, which can be calculated by Eq (17):

$$\begin{array}{c} \begin{array}{c} \Phi \left(F,r\right)=argmax\prod_{i=1}^{K}R_{exp}^{i}-v\chi_{i}\left({℧}_{i}\right) \end{array} \end{array}$$ (23)

Hence, we can use Eq (23), to confirm the selected solution for stable point in the previous section. This is also the point where “egalitarian” solutions of the game come in, and one such method is applicable to the equal gains principle, a Pareto optimal. For the 2 agents case in the previous section, the proportion *χ*_*i*_ in *Φ*, which is weakly efficient and satisfies the equal gain condition $\chi_{1}\left({℧}_{1}\right)-r_{1}=\chi_{2}\left({℧}_{2}\right)-r_{2}$, is called the “egalitarian” solution. As mentioned earlier, in our resource access method, the stable acts as a marketplace where the primary and secondary systems can do bargaining. The fair solution for two agents about one channel access is at the intersection of the egalitarian solutions:

$$\begin{array}{c} \begin{array}{c} \left\{\begin{array}{c} U_{1}-\chi_{1}\left({℧}_{1}\right)=U_{2}-R_{exp}^{2}\\ U_{1}+U_{2}=argmax_{\chi_{i}}\left(U_{1}^{\prime }+U_{2}^{\prime }\right) \end{array}\right. \end{array} \end{array}$$ (24)

Condition Eq (24) dictates that the operating point should be on the boundary of the minimum region. Therefore, the intersection gives the unique approximate fairness solution. For a *N* agents game, the fairness problem Eq (23), should be solved by calculating the *χ*_*i*_, *i* = 1, 2, …, *N*. To verify the case, we note that the stable point in the proposed design is also on the perpendicular boundary to (24) at its intersection. The corresponding optimization satisfies Eqs (15) and (17), defined by $\max_{\chi_{i}}\left[U_{1}-\chi_{1}\left({℧}_{1}\right)\right]\times \left[R_{exp}^{2}-U_{2}\right]$, which is subject to $\left(r_{i},B\right)$, *i* = 1, 2, …*N*. It is straightforward to confirm the solution of Eq (18), as it satisfies the description at the beginning of this section.

## Experiments and Results

In this section, we designed several experiments to evaluate the proposed methods in above sections. We employed the multi-agent platform in [4] to simulate the multi-agent Ad-hoc network. The data unit length was fixed at 1,024 bytes. We evaluate the performance of the proposed scheme with wide band available by simulation and compare it with the priced-based centralized channel allocation method (OPTIMAL) [26] and the RANDOM method to validate the efficiency, which allows agent randomly accesses channels from its current belief on each channel. The simulation parameters are shown in Table 2.

**Table 2:** Simulation parameters.

Simulation Parameters	Value
Number of agents	200–1000
Maximum number of channels	11
Number of perception channels	5
Number of access channels	3
Maximum resources for a channel	100
Data frame size	1

In order to facilitate the numerical statistics, we use one channel for global listening (especially for the OPTIMAL method of the centralized resource allocation), the rest of 10 channels allow agents to access. We conducted the simulations under various scale agents and the simulation results are the average value of 100 runs.

### Resource Lost Rate

**Fig 3:**
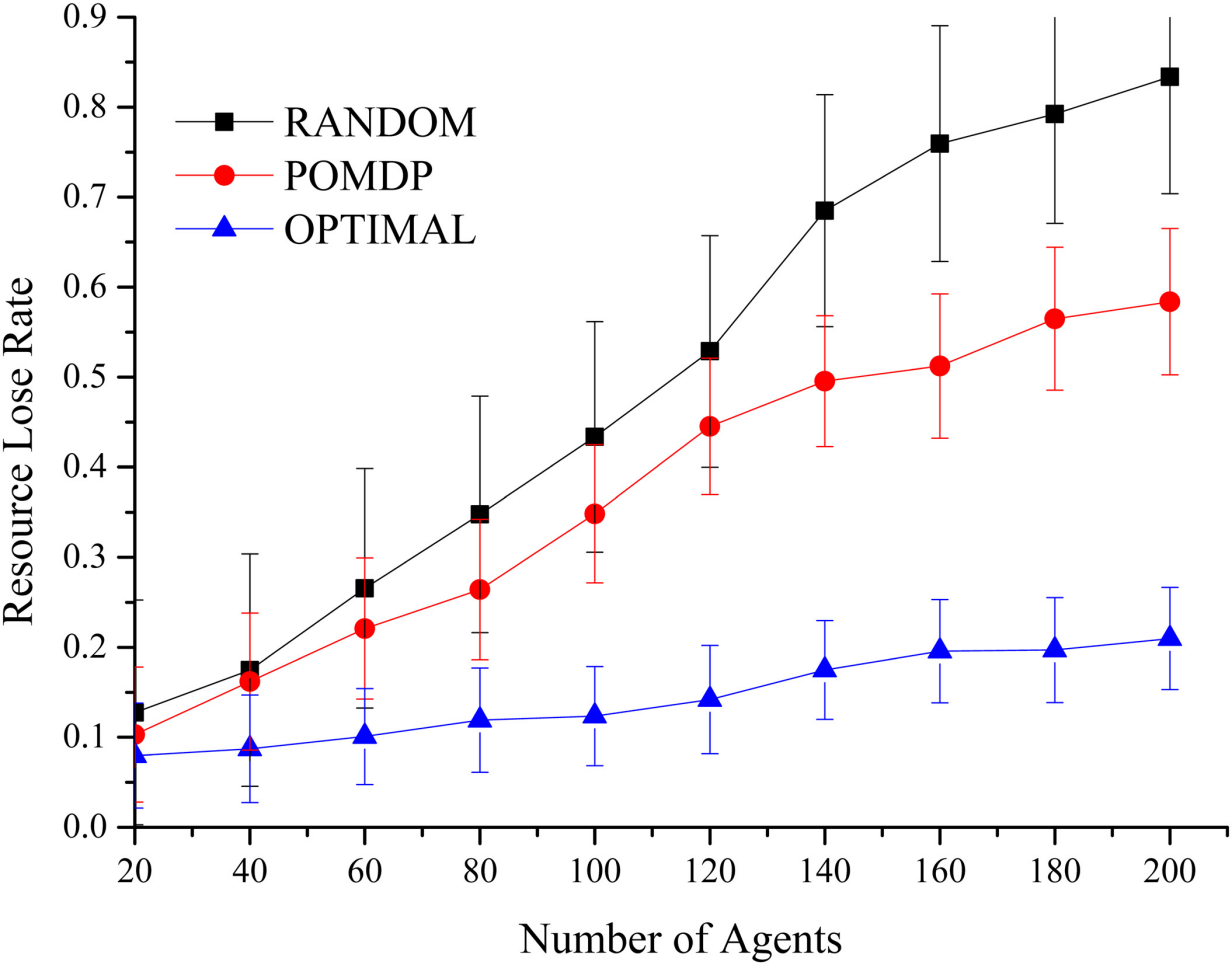
Resources Loss Rate. With the increased size of the agents, the randomness of the RANDOM method increased interference between agents, which brings down the network resources utilization. OPTIMAL method can maintain an efficient use of the resources, but its time consumption is much larger than the self-decision methods. POMDP methods maintain a relative balance to the above methods.

As in Fig 3, the results show that the influences of different channel access strategies, have direct impact on the available channel resources. The axes represent the number of agents and the resource loss rates. Agents adopt a RANDOM method, and with the expansion of scale (20–200, from 0 agent there has no conflict), the congestion and resource loss rates continue to rise closing to 90%. Meanwhile, in our algorithm, agents communicate with their neighbors to exchange decision policies, and acquire a better joint behavior through continuous negotiation and iterations (the average max amount of loss is 65.38%, the average sample variance is 8.12%), the variance shows that our algorithm is more stable.

The OPTIMAL method can provide a better result, but the resource consumption of global consultations could not be avoided (the average max amount of loss is 21.92%, the average sample variance is 5.67%). Furthermore, due to agents’ misperception and accessing, the resource loss (conflicts) is inevitable, and will increase sharply with the expansion of the agents.

### Resource Available Rate

**Fig 4:**
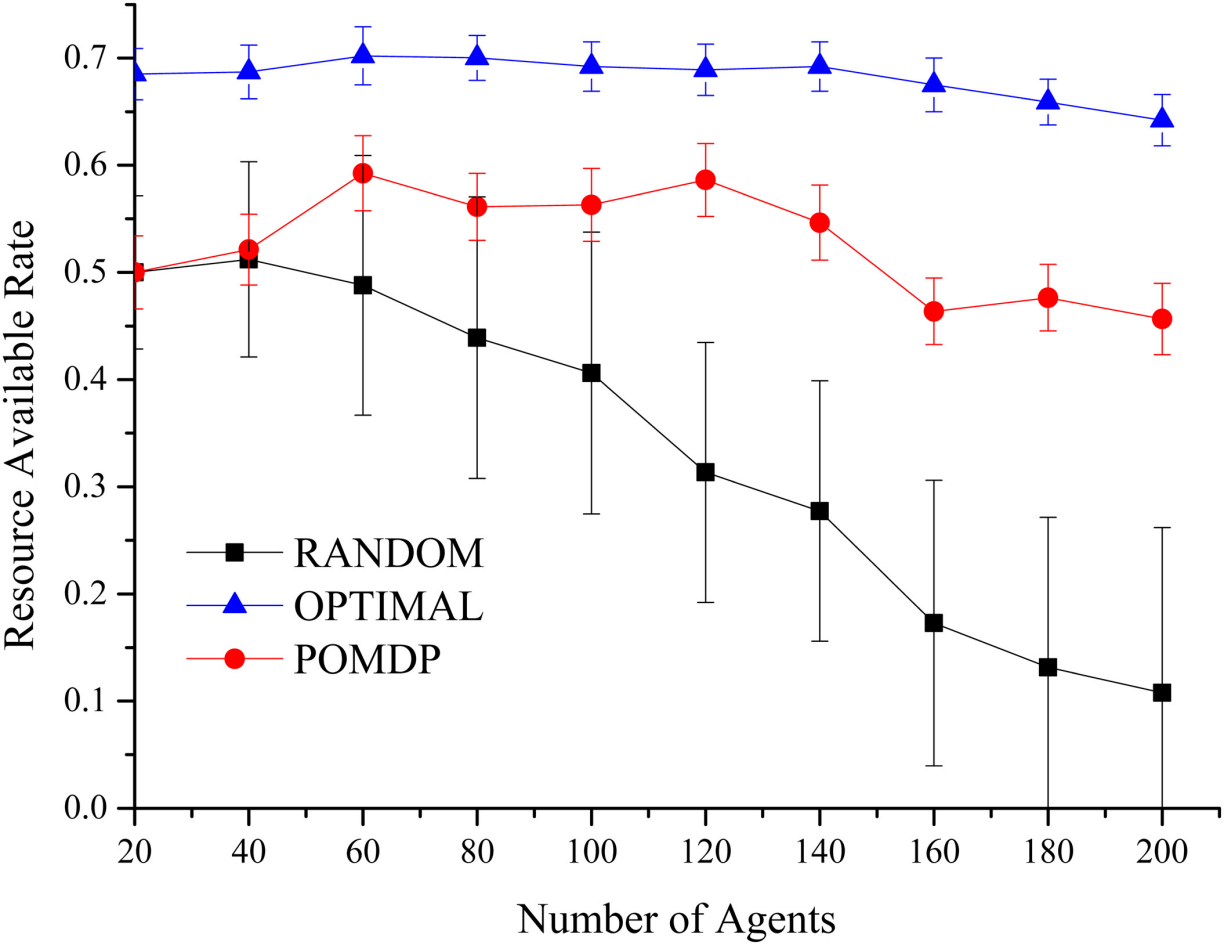
Resources Available Rate. Under the same experimental setup, with the increasing size of the agents, RANDOM method reduces the resources available for each agents. Because of neighbor’s awareness in POMDP, the agents can be maintained in a state of relative balance (less variance than RANDOM).

With the premise of partial observation, we set the RANDOM and our algorithm to start from the initial belief probability 0.5, as shown in Fig 4. But the difference is, our algorithm can reach an average resource showed at 52.67% and the average sample variance is 3.32%. RANDOM’s resource obtain rapid descent, and when there has 200 agents, the available resources only remain 33.48%, but with 12.16% average sample variance. When the number of agents and network resources are relatively homogeneous, the available resources rate can approach the expected value. Then due to the increase of the agents’ number, the available rates decrease. The OPTIMAL can keep an average resource obtained at 68.23% and with a very stable average sample variance 2.37%.

Obviously, agents can obtain more resources with our design than RANDOM provides. Especially, with the increase of the agents’ number and passing time (agents can exchange information with neighbors and accumulate from known knowledge), the resource availability rate remains in a relatively stable state until agents reaching network’s saturation.

### Available Resource in Different Interaction Frequencies

**Fig 5:**
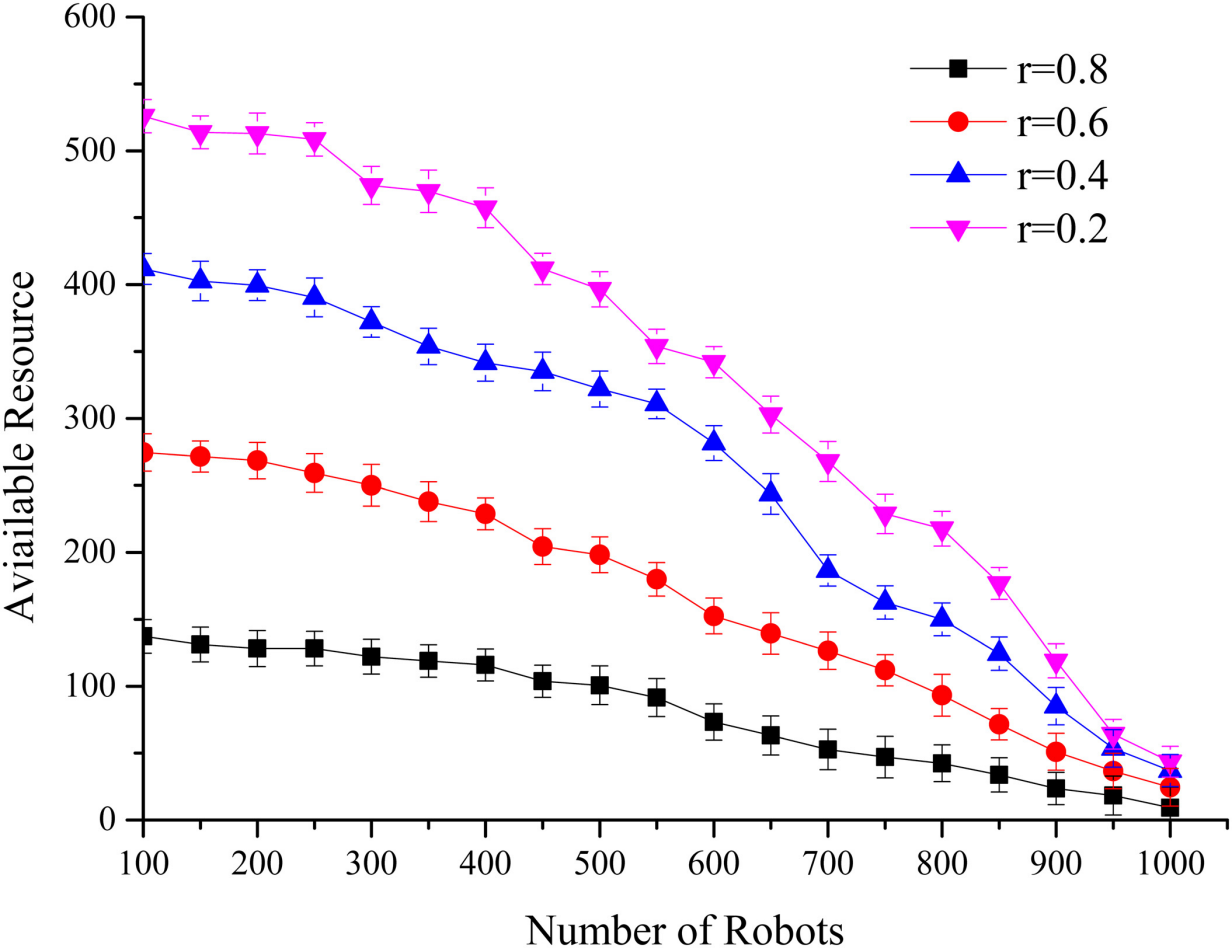
Available Resources in Different Interaction Frequencies. In different interaction frequencies, the available resources shrink with the increasing number of agents. Similar to the allocation of limited resources in human society, the average gain decreases with the increasing number of people. Experimental results are consistent with the general understanding.

In this simulation, we test the average available channel resources for the new accessed agents under different interaction frequencies of the other agents in the network. We set 5 channels and 100 per slot new accessed agents, which are uniformly distributed in these 5 channels, the max agents number is 1000. The interaction frequency of the other agents was set to *r* = [0.2, 0.4, 0.6, 0.8]. X-axis represents the agents’ number. As in Fig 5, we can find two significant changes for the new accessed agents: with increasing numbers of network agents or the higher interaction frequency, the available resources decline. In addition, when the number of agents is more than the maximum number the network can support, the available resources for the entire network will be sharply reduced.

According to the experiment’s results, we can make a bold hypothesis that while the number of agents and the resource relatively balance, there should be a suitable interaction frequency that makes each agent obtain available resources to maximize its utility.

### Resource Available in Different Assignments

**Fig 6:**
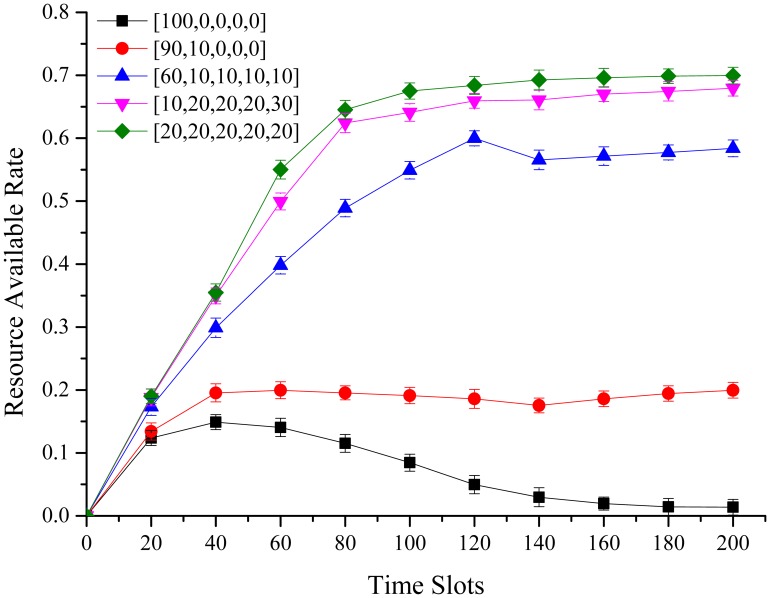
Resource Available in Different Assignments. In the 5 specified channel, the more uniform distribution of the agents, the higher the probability of their available resources, whereas the performance reduces (more crowded, no elimination of competition that makes the average income is lower).

In this simulation, we discuss the relationship between different team sizes when agents access channels under different assignment. We divide 100 agents into different team sizes, and allow them to access 5 channels. Simulation results are shown in Fig 6. Caused by new access agent, blocking rate of channel will increase and influence the original agents due to the partial observation.

Obviously, we can see that when the combination in each channel distribution is more uniform, the greater resource available, as assignment [10, 20, 20, 20, 30] and [20, 20, 20, 20, 20]. In an extreme access situation with [100, 0, 0, 0, 0], all agents are in one channel. When communication demand escalates, all agents almost have no chance to obtain available resources. From above analysis, we can conclude that agents will gain more available resources when they distribute more uniformly.

### Resource Available Comparison

**Fig 7:**
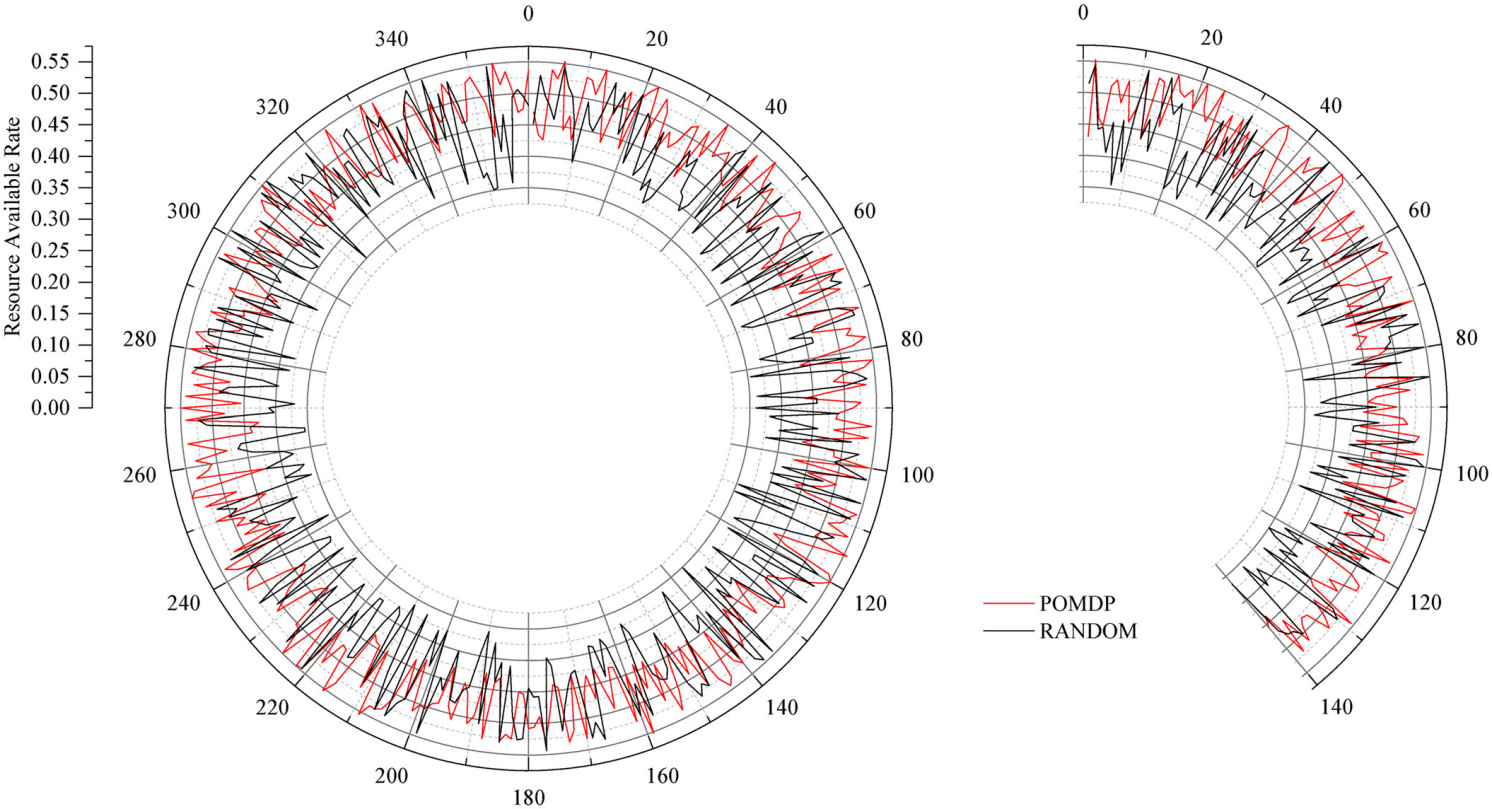
Resource Perception of 500 Tests. To illustrate the variances in the 4 aforementioned simulation results, this figure gives the resource perception comparison in 500 tests between POMDP and RANDOM.

Fig 7 displays the contrast of channel resources awareness between our algorithm and RANDOM. In this simulation, we set 100 agents with freedom interactive frequency.

It can be seen that, with 500 tests for the same channel (distribution within the circles), agents can obtain the actual state of the channel. The red trail denotes the search result by POMDP, and black trail denotes the RANDOM method. It is obvious that the randomness and divergence of RANDOM far outweigh that of POMDP.

## Conclusion

We assumed in this paper that channel state transition probabilities can be entirely perceived, but in practice, this may not be available. The problem then becomes a decision model with unknown transition probabilities, but such mode is beyond the scope of this paper. In our design, we reduce a Dec-POMDP model to a simplified one by separating the problem into single-agent decision coordination, which may result in a low-complexity but potentially suboptimal design. In practical applications, systems Dynamics making use of pure policy space searching to solve all the problems become impractical, and need to be adjusted according to the actual situation and dynamics, and add more factors. In our future work, we will pursue the optimal joint design of the tradeoff between complexity and optimality, and will apply reinforcement learning theory on real multi-robots platform.

## Supporting Information

S1 Table Experiment Data for Resource lost Rate Comparison. (XLS)

S2 Table Experiment Data for Resource Available Rate Comparison. (XLS)

S3 Table Experiment Data for Available Resource in Different Interaction Frequencies Comparison. (XLS)

S4 Table Experiment Data for Resource Available in Different Assignments Comparison. (XLS)

S5 Table Experiment Data for Available Resource Perception. (XLS)

S1 File Supplementary Methods and Datasets Introduction. Supporting Information Supplementary Methods and Datasets Introduction. (DOC)

## References

[1] Wang T, Dang Q, Pan P. A Multi-Robot System Based on A Hybrid Communication Approach. Studies in Media and Communication. 2013;1(1):91–100. 10.11114/smc.v1i1.124

[2] Iqbal, J, Yousaf, MM, Awais, MM, editors. A scalable approach of message interpretation by demonstrations for multi-robot communication. Multitopic Conference, 2009 INMIC 2009 IEEE 13th International; 2009: IEEE. 10.1109/INMIC.2009.5383082

[3] Conti M, Giordano S. Mobile ad hoc networking: milestones, challenges, and new research directions. Communications Magazine, IEEE. 2014;52(1):85–96. 10.1109/MCOM.2014.6710069

[4] Zhang Y, Xu Y, Hu H. Cooperative Decision Algorithm for Time Critical Assignment without Explicit Communication Intelligent Information Processing VII: Springer; 2014 p. 197–206.

[5] Liu M, Xu Y, Wu S, Lan T. Design and Optimization of Hierarchical Routing Protocol for 6LoWPAN. International Journal of Distributed Sensor Networks. 2015;2015 10.1155/2015/802387

[6] Ab Wahab MN, Nefti-Meziani S, Atyabi A. A Comprehensive Review of Swarm Optimization Algorithms. PLoS ONE. 2015;10(5): e0122827 10.1371/journal.pone.0122827 25992655 PMC4436220

[7] Zhao Q, Sadler BM. A survey of dynamic spectrum access. Signal Processing Magazine, IEEE. 2007;24(3):79–89. 10.1109/MSP.2007.361604

[8] Kulkarni RV, Venayagamoorthy GK. Particle swarm optimization in wireless-sensor networks: A brief survey. Systems, Man, and Cybernetics, Part C: Applications and Reviews, IEEE Transactions on. 2011;41(2):262–7. 10.1109/TSMCC.2010.2054080

[9] Yan Z, Jouandeau N, Cherif AA. A survey and analysis of multi-robot coordination. International Journal of Advanced Robotic Systems. 2013;10:399 10.5772/57313

[10] Capitan J, Spaan MT, Merino L, Ollero A. Decentralized multi-robot cooperation with auctioned POMDPs. The International Journal of Robotics Research. 2013;32(6):650–71. 10.1177/0278364913483345

[11] Tan L, Feng Z, Li W, Jing Z, Gulliver TA. Graph coloring based spectrum allocation for femtocell downlink interference mitigation. Wireless Communications and Networking Conference (WCNC), 2011 IEEE; 2011: IEEE. 10.1109/WCNC.2011.5779338.

[12] Xu Y, Wang J, Wu Q, Anpalagan A, Yao Y-D. Opportunistic spectrum access in cognitive radio networks: Global optimization using local interaction games. Selected Topics in Signal Processing, IEEE Journal of. 2012;6(2):180–94. 10.1109/JSTSP.2011.2176916

[13] Tang S, Mark BL, editors. Performance analysis of a wireless network with opportunistic spectrum sharing. Global Telecommunications Conference, 2007 GLOBECOM’07 IEEE; 2007: IEEE. 10.1109/glocom.2007.880.

[14] Liu H, Krishnamachari B, Zhao Q, editors. Cooperation and learning in multiuser opportunistic spectrum access. Communications Workshops, 2008 ICC Workshops’ 08 IEEE International Conference on; 2008: IEEE. 10.1109/ICCW.2008.98.

[15] Busoniu L, Babuska R, De Schutter B. A comprehensive survey of multiagent reinforcement learning. Systems, Man, and Cybernetics, Part C: Applications and Reviews, IEEE Transactions on. 2008;38(2):156–72. 10.1109/TSMCC.2007.913919

[16] Fernandez-Gauna B, Graña M, Lopez-Guede JM, Etxeberria-Agiriano I, Ansoategui I. Reinforcement Learning endowed with safe veto policies to learn the control of Linked-Multicomponent Robotic Systems. Information Sciences. 2015;317:25–47.

[17] Fernandez-Gauna B, Etxeberria-Agiriano I, Graña M. Learning Multirobot Hose Transportation and Deployment by Distributed Round-Robin Q-Learning. PLoS ONE. 2015;10(7): e0127129 10.1371/journal.pone.0127129 26158587 PMC4497621

[18] Halldórsson MM, Halpern JY, Li LE, Mirrokni VS, editors. On spectrum sharing games. Proceedings of the twenty-third annual ACM symposium on Principles of distributed computing; 2004: ACM. 10.1145/1011767.1011783.

[19] Yichen W, Pinyi R, Zhou S. A POMDP based distributed adaptive opportunistic spectrum access strategy for cognitive ad hoc networks. IEICE transactions on communications. 2011;94(6):1621–4.

[20] Seuken S, Zilberstein S. Improved memory-bounded dynamic programming for decentralized POMDPs. arXiv preprint arXiv:12065295. 2012.

[21] Feng M, Qu H, Yi Z. Highest Degree Likelihood Search Algorithm Using a State Transition Matrix for Complex Networks. Circuits and Systems I: Regular Papers, IEEE Transactions on. 2014;61(10):2941–50. 10.1109/TCSI.2014.2333677

[22] Kish LB, Harmer GP, Abbott D. Information transfer rate of neurons: stochastic resonance of Shannon’s information channel capacity. Fluctuation and Noise Letters. 2001;1(01):L13–L9. 10.1142/S0219477501000093

[23] Niyato D, Hossain E, Han Z. Dynamics of multiple-seller and multiple-buyer spectrum trading in cognitive radio networks: A game-theoretic modeling approach. Mobile Computing, IEEE Transactions on. 2009;8(8):1009–22. 10.1109/TMC.2008.157

[24] Hansen E A, Bernstein D S, Zilberstein S. Dynamic programming for partially observable stochastic games. AAAI. 2004;4:709–715.

[25] Roca CP, Cuesta JA, Sánchez A. Evolutionary game theory: Temporal and spatial effects beyond replicator dynamics. Physics of life reviews. 2009;6(4):208–49. 20416850 10.1016/j.plrev.2009.08.001

[26] Xue Y, Li B, Nahrstedt K. Optimal resource allocation in wireless ad hoc networks: A price-based approach. Mobile Computing, IEEE Transactions on. 2006;5(4):347–64. 10.1109/TMC.2006.1599404

