# Supplementary material for: Decentralized Opportunistic Spectrum Resources Access Model and Algorithm toward Cooperative Ad-Hoc Networks
Source: PLoS One. 2016 Jan 4;11(1):e0145526. doi: 10.1371/journal.pone.0145526 (PMC4699651; doi:10.1371/journal.pone.0145526)
Supplement: S1 File — (DOC) [file pone.0145526.s006.doc]

**Supplementary Methods and Datasets Introduction**

**1 PURPOSE AND SCOPE**

In this document, the first part will introduce several fundamental methods and theories we applied in our research but not into details. The other part describes the datasets of our simulation results.

**2 METHODOLOGY**

**2.1 OPTIMAL METHOD**

**-Dynamic Equilibrium Resource Allocation**

We consider a dynamic resource allocation model where agents arrive at different times and do not depart. For instance, we assume that agent 1 arrives first before agent 2, and in general, agent k arrives after agents 1,...,k-1; we say that agent k arrives in step k. An agent reports its demand when it arrives and the demand does not change over the time step. Thus, at step k, demand d_k_ is known, and demand d_>k_ is unknown. The dynamic resource allocation mechanism operates as follows: at each step k, the mechanism takes as input the reported demand d_k_ and outputs an allocation A_k_ over the agents present in the system. We also assume that the mechanism knows the total number of agents n in advance.

Existing work on static resource allocation focused on designing mechanisms that satisfy three prominent desiderata, but they can extend to the dynamic setting.

1. When an agent arrives, it receives an allocation that it likes at least as much as an equal split of the resources. This models a setting where agents have made equal contributions to the system, and hence have equal entitlements. In such cases, the contributions are typically recorded, which allows the mechanism to know the total number of agents n in advance, as assumed in our setting.
2. A dynamic allocation mechanism is that no agent can misreport its demand and be strictly better off at any step k, regardless of the reported demands of other agents. Formally, a dynamic allocation mechanism is SP if for any agent i∈N and any step k, if A_k_ is the allocation to agent i at step k when agent i reports its true demand and B_k_ is the allocation to agent i at step k when agent i reports a different demand (in both cases all the other agents report their true demand vectors), then u_i_(A_k_)>u_i_(B_k_). The design avoids introducing additional notations that will not be required later.
3. A dynamic allocation mechanism is in equilibrium if at each step k, the allocation A_k_ returned by the mechanism is not Pareto dominated by any other allocation B_k_ that allocates up to a (k/n)-fraction of each resource among the k agents present in the system. Put another way, at each step the allocation should not be Pareto dominated by any other allocation that only redistributes the collective entitlements of the agents present in the system among those agents.

In a static setting, the three prominent axiom means that the mechanism’s allocation is not Pareto dominated by any other allocation. Of course, in the dynamic setting it is unreasonable to expect the allocation in early stages to be Pareto abominated, because we need to save resources for future arrivals (recall that allocations are irrevocable).

**2.2 POMDP SEARCH METHOD**

**POMDP**

**-General Model Definition**

POMDPs address the problem of selecting actions in stationary, partially observable, controllable Markov chains. This is defined as:

- State - at any point in time, the world is in a specific state, denoted by *x*.
- Action - the agent can execute actions, denoted by *a*.
- Observation - through its sensors, the agent can observe a (noisy) projection of the world’s state. We use *Ω* to denote observations.
- Reward - additionally, the agent receives rewards/penalties, denoted by *R*. To simplify the notation, we assume that the reward is part of the observation. More specifically, we will use *R(o)* to denote the function that “extracts” the reward from the observation.

A POMDP can characterized by three probability distributions:

1. The initial distribution, π(x):= Pr(x_0_), specifies the initial distribution states at time t=0.
2. The next state distribution, μ(x’|a, x):= Pr(x_t_=x’|a_t-1_= a, x_t-1_= x), describes the likelihood that action a, when executed at state x, leads to state x’.
3. The perceptual distribution, υ(o|x):=Pr(o_t_=o|x_t_=x), describes the likelihood of observing o when the world is in state x.

The fundamental problem in POMDP is to devise a policy for action selection that maximizes reward. A policy, denoted by: π: o→a is a mapping from observation to action. Each policy induces an expected cumulative and possibly discounted by a discount factor reward.

**Monte-Carlo Tree Search**

Monte-Carlo Tree search (MCTS) uses Monte-Carlo method to evaluate the nodes of a search tree in a sequentially best-first order. There is one node in the tree for each state *s*, containing a value Q(s,a) and a visitation count N(s,a) for each action a, and an overall count N(s)=Pa, N(s,a). Each node is initialized to Q(s,a)=0, N(s,a)=0. The value is estimated by the mean return from s of all simulations in which action a was selected from state s. Each simulation starts from the current state st, and is divided into two stages: a tree policy that is used while within the search tree; and a roll out policy that is used once simulations leave the scope of the search tree. The simplest version of MCTS uses a greedy tree policy during the-first stage, which selects the action with the highest value; and a uniform random roll out policy during the second stage.

After each simulation, one new node is added to the search tree, containing the-first state visited in the second stage. Each state of the search tree is viewed as a multi-armed bandit, and actions are chosen by using the decision algorithm. The value of an action is augmented by an exploration bonus that is highest for rarely tried actions. The scalar constant c determines the relative ratio of exploration to exploitation. Once all actions from state s are represented in the search tree, the tree policy selects the action maximizing the augmented action-value, *argmax*_a_Q(s,a). Otherwise, the roll out policy is used to select actions. For suitable choice of c, the value function constructed converges in probability to the optimal value function, Q(s,a) →Q*(s,a). New nodes are initialized using this knowledge, Q(s,a)=Q_init_(s,a), N(s,a)=N_init_, where Q_init_(s,a) is an action value function and N_init_ indicates its quality. Domain knowledge narrowly focuses the search on promising states without altering asymptotic convergence.

**2.3 STOCHASTIC APPROXIMATION**

**RANDOM METHOD**

Consider the problem of trying to find the approximate value based on noise-free measurements. Random search methods are perhaps the simplest methods of stochastic optimization in such a setting, and can be quite effective in many problems. Their relative simplicity is an appealing feature to both practitioners and theoreticians. These direct random search methods have a number of advantages relative to most other search methods. The advantages include relative ease of coding in software, the need to only obtain measurements (versus gradients or other ancillary information), reasonable computational efficiency (especially for those direct search algorithms that make use of some local information in their search), broad applicability to non-trivial loss functions and/or that may be continuous, discrete, or some hybrid form, and a strong theoretical foundation.

A simple, but very popular approach is the random method, which centers a symmetric probability density function (e.g., the normal distribution), about the current best location. The standard normal N(0,1) is a popular choice, although the uniform distribution U[-1,1] is also common. A variation of the random method determines the maximum of the objective function by analyzing the distribution of J(v) in the bounded sub-region. In this variation, the random data are fitted to an asymptotic extreme-value distribution, and J* is estimated with a confidence statement. Unfortunately, these techniques cannot determine the location of J*, which can be as important as the J value itself. Some techniques calculate the mean value and the standard deviation of J(v) from the random data as they are collected.

**Localized Random Search (Abstract Method)**

- Step 0

(Initialization) Pick an initial guess Θ_0_∈O, either randomly or with prior information. Set k=0.

- Step 1

Generate an independent random vector dk∈R and add it to the current O value, Θ_k_. Check if Θ_k_+d_k_∈O. If Θ_k_+d_k_ does not belong to O, generate a new d_k_ and repeat, or alternatively, move Θ_k_+d_k_ to the nearest valid point within O. Let Θ_new_(k+1) equal Θ_k_+d_k_∈O or the aforementioned nearest valid point in O.

- Step 2

If L(Θ_new_(k+1)) < L(Θ_k_), set Θ_k+1_=Θ_new_(k+1); else, Θ_k+1_=Θ_k_.

- Step 3

Stop if the maximum number of L evaluations has been reached or the user is otherwise satisfied with the current estimate for O via appropriate stopping criteria; else, return to Step 1 with the new k set to the former k+1.

For continuous problems, some researches have used the (multivariate) normal distribution for generating d_k_. The distribution should have mean zero and each component should have a variation (e.g., standard deviation) consistent with the magnitudes of the corresponding O elements. This allows the algorithm to assign roughly equal weight to each of the components of O as it moves through the search space. Although not formally allowed in the convergence theory, it is often advantageous in practice if the variability in d_k_ is reduced as k increases. This allows one to focus the search more tightly as evidence is accrued on the location of the solution (as expressed by the location of our current estimation).

**2.4 GAME METHOD**

**Implicit Dynamic Pareto Optimal**

Considering n agents arriving in the system and then using a resource allocation mechanism. However, this scheme is highly inefficient, e.g., it is easy to see that one can always allocate each agent at least a 1/n share of its dominant resource (and other resources in proportion) as soon as it arrives and still maintain equilibrium at every step. Given a step k∈{1,...,n}, define an allocation A over the k present agents with demands d_<k_ to be equilibrium if it can be extended to an equilibrium allocation over all n agents with demands d=(d_<k_, d_>k_), for all possible future demand.

*Implicit Dynamic Pareto Optimal (IDPO).* A limit resource dynamic allocation mechanism satisfies ICPO if at every step k, the allocation A_k_ returned by the mechanism is not Pareto dominated by any other allocation A’ over the same k agents that is dynamic equilibrium. In other words, a mechanism satisfies ICPO if at every step it selects an allocation that is at least as generous as any allocation that can ultimately guarantee equilibrium, irrespective of future demands. At first glance, it may not be obvious that ICPO is indeed a relaxation of DPO (i.e., that ICPO is implied by DPO). However, note that DPO requires a mechanism to allocate at least a k/n fraction of at least one resource r* in the allocation A_k_ at any step k, and thus to allocate at least a 1/n fraction of that resource to some agent i. Any alternative allocation that Pareto dominates A_k_ must also allocate at least a 1/n fraction of r* to agent i. Consequently, in order to ensure an equilibrium over all n agents when all the future demands are identical to the demand of agent i, the alternative allocation must allocate at most a k/n fraction of r*, as each future agent may also require at least a 1/n fraction of r* to avoid envying agent i. It follows that the alternative allocation cannot Pareto dominate A_k_. Thus, the mechanism satisfies ICPO.

We wish to extend ICPO to focus only on competing with equilibrium allocations. The main technical challenge is checking when an allocation at step k violates equilibrium. Indeed, there are uncountable many possibilities for the future demands d_>k_ over which an equilibrium extension needs to be guaranteed by an equilibrium allocation. Of course, checking all the possibilities explicitly is not feasible. Ideally, we would like to check only a small number of possibilities.

**3 DATA SET INSTRUCTION**

In each excel file “Si Table.xls” file, i∈(1,2,3,4,5), we provide one sample o raw data and the data for figures of the simulation. The average data points, and measurement deviations used in generating the figures of this paper are provided under each simulation results (generated from multiple test results, not only from the above sample).

In each figure, the 10 data points considered correspond to the average values of each data column over 100 runs for each simulation.
